# Deterministic assembly and centralized networks define the *Pinus massoniana* rhizosphere mycobiota

**DOI:** 10.3389/fpls.2026.1844534

**Published:** 2026-06-18

**Authors:** Hongjuan Wang, Mingyuan Zhang, Lirui Chen, Jiani Lin, Shaolong Li, Liang Zhao, Pengpeng Dou

**Affiliations:** 1 Chongqing Key Laboratory of Adversity Agriculture Research, Chongqing Academy of Agricultural Sciences, Chongqing, China; 2 Key Laboratory of the Three Gorges Reservoir Region’s Eco-Environment, Ministry of Education, Chongqing University, Chongqing, China; 3 Institute of Desertification Studies, Institute of Ecological Conservation and Restoration, Chinese Academy of Forestry, Beijing, China

**Keywords:** community assembly, co-occurrence network, fungal community, Pinus massoniana, rhizosphere effect

## Abstract

**Introduction:**

The rhizosphere serves as the most active interface for plant–soil microbial interactions. Resource inputs and micro-environmental gradients within this zone are expected to drive the selective recruitment and structural reorganization of fungal communities. However, systematic evidence regarding the divergence patterns and assembly mechanisms between rhizosphere and bulk soil fungal communities in coniferous forests in subtropical area remains limited.

**Methods:**

In this study, we collected 44 soil samples (rhizosphere and bulk soil) from *Pinus massoniana* plantations. Fungal amplicon sequencing was conducted, integrated with indicator taxa analysis, *β*-diversity comparisons, null model frameworks (Normalized Stochasticity Ratio, NST), and co-occurrence network topological analysis to resolve the fungal communities “composition–assembly–structure” variations across these two microhabitats.

**Results:**

A total of 2,398 fungal ASVs were identified. While the majority of taxa were shared between habitats, the community composition exhibited significant differentiation: the rhizosphere was relatively enriched with Ascomycota and Mortierellomycota, whereas Basidiomycota predominated in the bulk soil. Multiple potential bioindicators were identified for each niche. Although α-diversity indices showed no significant differences between microhabitats, *β*-diversity revealed a distinct separation in community structure, and with higher inter-sample heterogeneity observed in the rhizosphere. NST analysis indicated that fungal assembly in the rhizosphere was predominantly governed by deterministic processes, while stochastic processes played a more dominant role in the bulk soil. Co-occurrence networks further demonstrated that both microhabitats possessed highly modular structures. However, the bulk soil network was more complex and highly connected, whereas the rhizosphere network was more centralized with a more uneven distribution of nodal connections.

**Discussion:**

These findings provide comparative evidence for the divergence in fungal community assembly and network organization in subtropical coniferous forests, offering valuable insights for the assessment and management of forest soil health from a microbial perspective.

## 1 Introduction

The rhizosphere represents the most biologically active interface between plant roots and soil. Often characterized as the plant’s “second genome”, the rhizosphere microbiome regulates plant health and ecosystem processes through nutrient mobilization, pathogen antagonism, and organic matter transformation (Berendsen et al., 2012; Philippot et al., 2013). Within this microhabitat, the spatiotemporal dynamics of root exudates provide periodic pulses of carbon and act as signaling cues (Oppenheimer-Shaanan et al., 2022). These exudates engage in “metabolic coupling” with the substrate preferences of specific microorganisms, driving rapid community reorganization and directional enrichment in the vicinity of the root surface (Zhalnina et al., 2018). Consequently, rhizosphere-driven divergence in microbial communities often manifests not as a simple increase or decrease in species richness, but rather as the replacement of dominant taxa, niche differentiation, and the reassembly of functional units (Philippot et al., 2013). The bulk soil matrix represents a relatively quiescent, mineral-dominated background that functions as the primary regional species pool, where microbial assembly is broadly governed by ambient soil physicochemical properties and stochastic dispersal limitation. Conversely, the rhizosphere constitutes a highly dynamic plant–microbe interface receiving continuous inputs of host-derived organic carbon, thereby imposing an intense localized selective filter.

In forest ecosystems, fungi are key drivers of belowground processes, with their diversity and biogeographic patterns exhibiting strong environmental dependence (Tedersoo et al., 2014). In coniferous forests, extensive evidence supports the critical roles of ectomycorrhizal fungi—predominantly within the Basidiomycota—in nitrogen and phosphorus acquisition and carbon sequestration (Clemmensen et al., 2015). However, a vast array of non-mycorrhizal fungi, such as saprotrophic and endophytic groups within the Ascomycota, also colonize the rhizosphere and endosphere. Despite their participation in organic matter decomposition, nutrient mineralization, and the construction of microbial interaction networks, these groups are frequently less emphasized than “mycorrhizal-centric” narratives (Tedersoo et al., 2014). Furthermore, the woody plant rhizosphere often exhibits a divergence where strong host selection coexisting with high community heterogeneity: while host-associated “hub taxa” may persist across large spatial scales, local communities remain highly variable (Toju et al., 2018). Thus, deciphering forest rhizosphere fungal communities requires moving beyond describing taxonomic shifts to addressing the underlying assembly process—specifically, whether root-driven environmental filtering enhances deterministic processes at the expense of stochasticity and how this alters the network organization strategies of the community.

From a community ecology perspective, the divergence between the rhizosphere and bulk soil can be understood as a shift in the relative weight of deterministic and stochastic processes. Rooted in traditional niche theory, deterministic processes refer to non-random, environmentally or biologically driven mechanisms—such as environmental filtering, host selection, and interspecific competition—that shape community structures toward predictable outcomes based on organismal fitness variations (Zhou and Ning, 2017). Conversely, stochastic processes are governed by neutral dynamics, including dispersal limitation, ecological drift, and random birth-death events (Jiang et al., 2025). In forest soils characterized by complex physical structures and significant micro-environmental patchiness, the relative contribution of these processes remains inconsistent and requires quantitative validation using null model frameworks and stochasticity metrics (Zhou and Ning, 2017). Importantly, previous studies in the same *Pinus massoniana* plantation system have shown that bulk soil fungal communities are associated with edaphic variables, including soil bulk density, electrical conductivity, and available phosphorus (Chen et al., 2022), whereas rhizosphere fungal divergence is more closely related to root traits than to soil physicochemical properties (Lin et al., 2024). This contrast suggests that fungal community organization may be governed by different ecological filters between the bulk soil and the rhizosphere: broad edaphic filtering in the bulk soil matrix and stronger host-mediated selection in the rhizosphere. Concurrently, co-occurrence networks offer a systemic perspective for characterizing community organization. Emerging research suggests that microbial diversity and network complexity and interconnectivity jointly influence ecosystem multifunctionality, while the network’s response to perturbations may dictate community vulnerability and recovery trajectories (Chen et al., 2023; de Vries et al., 2018; Li and Wu, 2025). Therefore, it is insufficient to merely describe “which taxa are present”; one must also employ network topology to understand how these taxa are organized. It is important to emphasize that while such correlation-based networks do not directly demonstrate biological interactions or causality, topological attributes such as connectivity, centralization, and modularity serve as indirect proxies for understanding community organization strategies and potential robustness (Olesen et al., 2007).

In this study, we investigated *Pinus massoniana* plantations—the most widely distributed plantation type in Southern China—to characterize the divergence of fungal communities between rhizosphere and bulk soils. Building upon our prior findings which established the environmental drivers in these plantations—where soil physicochemical properties predominantly modulate bulk soil matrix variation while root traits uniquely govern rhizosphere fungal divergence (Chen et al., 2022; Lin et al., 2024)—this study further resolves the intrinsic biological assembly rules and network interaction topologies of the mycobiota itself. By integrating high-throughput sequencing, null model frameworks, and co-occurrence network analysis, we tested the following hypotheses: (1) Significant taxonomic divergence exists between the two microhabitats, with the rhizosphere selectively enriching specialized fungal taxa capable of rapidly responding to root-derived carbon inputs; (2) Community assembly mechanisms differ between compartments; specifically, driven by intense host selection and stable resource pulses, deterministic processes exert a dominant influence in the rhizosphere, whereas bulk soil assembly is more susceptible to stochastic processes, such as dispersal limitation and ecological drift; (3) Rhizosphere resource gradients and microhabitat heterogeneity result in a more centralized and modular co-occurrence network compared to bulk soil. Conversely, the bulk soil network, while potentially exhibiting high connectivity, lacks the biotic buffering of the host and is thus more sensitive to environmental perturbations. By integrating these biological organization patterns with established edaphic baselines, this study provides empirical insights into how forest trees functionally modulate belowground mycobiota.

## 2 Material and methods

### 2.1 Study area

**Figure 1:**
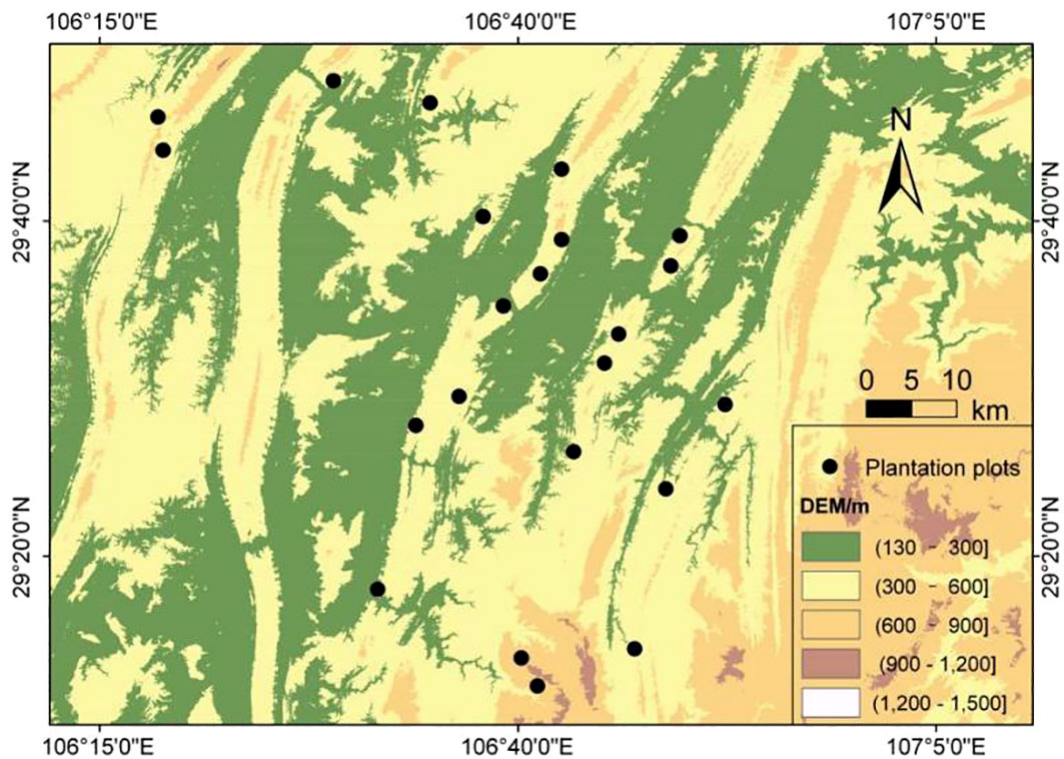
Spatial distribution of the 22 *Pinus massoniana* plantation plots in this study.

This study was conducted in the “Four Mountains” area (i.e., Jinyun, Zhongliang, Tongluo, and Mingyu Mountains) of Chongqing’s main urban area (106°14′36″–106°59′ 58″ E, 29°17′45″–30°7′22″N; **Figure 1**). The climate is a typical subtropical monsoon climate, with a mean annual temperature of 16–18 °C and a mean annual precipitation of 1,000–1,350 mm. The vegetation is characterized by high coverage of forests, including primary remnants, secondary regrowth evergreen broad-leaved forests, and pine plantations (Luo et al., 2020).

From September to December 2020, 22 representative *P. massoniana* plantation plots (20 m × 20 m) were established (**Figure 1**). For each plot, geographical parameters including coordinates, elevation, slope, and aspect were recorded. Each plot was further divided into sixteen 5 m×5 m subplots. All woody individuals with a diameter at breast height≥3 cm and height≥1.3 m were tagged and measured

### 2.2 Soil sample collection and processing

In December 2020, 12 sampling points were established within each plot along an *S*-shaped transect, with a minimum spacing of 3 m between adjacent points. After removing the surface litter layer, mineral soil samples were collected from a depth of 0–10 cm using a 32 mm inner diameter soil auger. A total of 12 cores per plot were pooled into a single sterile self-sealing bag and thoroughly homogenized to form a composite sample. Samples were immediately placed in a portable cooler with ice packs and transported to the laboratory. In the lab, approximately 10 g of the homogenized soil was subsampled using a sterile spoon, transferred into a small sterile bag, and stored at -80 °C for subsequent microbial community analysis. This procedure yielded 22 independent bulk soil composite samples representing the 22 study plots.

For rhizosphere soil sampling, six healthy *P. massoniana* individuals were randomly selected per plot. After clearing surface vegetation and litter, fine roots (1st–3rd order) were excavated at a depth of 0–10 cm within a 1-m radius of the trunk; roots were manually traced back to the primary roots to ensure host specificity. Rhizosphere soil was separated following the protocol of McPherson et al. (2018). Briefly, roots were gently agitated to remove loosely adhering bulk soil, and the remaining root segments with tightly adhering soil were immediately placed into 50-mL sterile tubes containing a phosphate buffer (6.33 g/L K_2_HPO_4_, 8.5 g/L KH_2_PO_4_, and 200 μL/L Silwet L-77; pH 6.5). Samples were transported to the laboratory in cold storage (4 °C). To dislodge the rhizosphere soil, tubes were vortexed for 2 min, after which the roots were removed with sterile forceps. The resulting suspension was filtered through a 100-μm sterile mesh and centrifuged at 3,000× g for 5 min. The pellet was then resuspended in 1.5 mL of sterile buffer and centrifuged at 15,871× g for 2 min. The final rhizosphere soil pellets were stored at -80 °C for subsequent DNA extraction and microbial analysis, resulting in 22 corresponding rhizosphere composite samples.

### 2.3 Molecular analyses

Total genomic DNA was extracted from 0.3 g of homogenized soil (from both rhizosphere and bulk soil) using the FastDNA^®^ SPIN Kit for Soil (MP Biomedicals, USA). DNA quality and quantity were assessed using a NanoDrop 2000 spectrophotometer and 1% agarose gel electrophoresis. The fungal ITS2 region was amplified using the universal primers ITS3F (5’-GCATCGATGAAGAACGCAGC-3’) and ITS4R (5’-TCCTCCGCTTATTGATATGC-3’) (Toju et al., 2012). Each 20-μL PCR reaction contained 4 μL of 5 × TransStart FastPfu buffer, 2 μL of dNTPs (2.5 mM), 0.4 μL of TransStart FastPfu DNA polymerase, and 10 ng of template DNA. The thermal cycling conditions involved an initial denaturation at 95 °C for 3 min, followed by 35 cycles of 95 °C for 30 s, 55 °C for 30 s, and 72 °C for 45 s, with a final extension at 72 °C for 10 min. PCR products were pooled in triplicate, purified using an agarose gel DNA purification kit, and prepared for sequencing using the NEXTflex™ Rapid DNA-Seq Kit. The concentration of the purified amplicons was quantified using a Qubit 2.0 Fluorometer (Thermo Fisher Scientific, USA), and library integrity was verified using an Agilent 2100 Bioanalyzer (Agilent Technologies, USA). Subsequently, finalized amplicon libraries were pooled in equimolar amounts and subjected to paired-end sequencing (2 × 250 bp) on an Illumina NovaSeq 6000 platform according to standard manufacturer workflows. Raw sequences are available in the NCBI SRA (PRJNA732174 and PRJNA905526 for bulk soil and rhizosphere fungi, respectively).

### 2.4 Bioinformatic analyses

The raw bidirectional sequence data were processed through a stringent quality-control and assembly pipeline. Initial quality filtering of the raw FASTQ files was performed using fastp (version 0.19.6). Specifically, trailing bases with a quality score below 20 were trimmed. A 10-bp sliding-window approach was implemented, whereby the remaining downstream read was truncated from the start of the window if the average quality score within that window fell below 20. Following this quality trimming, reads shorter than 50 bp or those containing more than 5 ambiguous bases (N) were strictly discarded. Paired-end reads were subsequently merged into longer single composite sequences using FLASH (version 1.2.11) based on their overlapping regions, requiring a minimum overlap length of 10 bp. A maximum mismatch ratio of 0.2 was permitted within the overlapping region, and any merged sequences exceeding this threshold were excluded. Individual samples were then demultiplexed and partitioned into their respective profiles based on their unique sample-specific barcodes and primer sequences, concurrently adjusting the sequence orientation to the standard forward direction. This demultiplexing enforced complete fidelity for barcodes (0 mismatches allowed) and permitted a maximum of 2 mismatches for the universal primers. The resulting high-quality merged sequences were then introduced into the DADA2 plugin within the QIIME 2 (version 2020.2) pipeline for denoising. Taxonomic assignment was performed using a Naive Bayes classifier trained on the UNITE Version 8.0 database with a confidence threshold of 0.7. As a global quality control baseline for all downstream analyses (including diversity, composition, and assembly profiling), ASVs with a relative abundance < 0.0005% or those assigned to chloroplasts and mitochondria were excluded.

### 2.5 Statistical analysis

Venn diagrams were constructed to visualize the distribution of unique and shared fungal taxa at the phylum, order, genus, and ASV levels between the rhizosphere and bulk soil microhabitats. The Chao1 richness, Shannon diversity, Pielou’s evenness, and Phylogenetic Diversity (PD) index were calculated to characterize the α-diversity of fungal communities in rhizosphere and bulk soils. Significant differences in alpha diversity between the paired rhizosphere and bulk soil samples were evaluated using paired Wilcoxon signed-rank tests. Differences in the relative abundance of fungal taxa at the phylum level were assessed. Linear discriminant analysis (LDA) effect size (LEfSe) was employed to identify significant biomarkers enriched in the rhizosphere and bulk soil habitats, with criteria set at a *P* < 0.05 (Kruskal–Wallis test) and an LDA score > 4. Fungal community *β*-diversity was visualized via Principal cooradinates analysis (PCoA) based on Bray–Curtis distances, and the statistical significance of community dissimilarity between habitats was tested using Permutational multivariate analysis of variance (PERMANOVA). To quantify the underlying mechanisms of fungal community assembly, the Normalized Stochasticity Ratio (NST) was calculated based on a null model framework using the “NST” package in R (Ning et al., 2019). We utilized the Bray–Curtis distance metric and the “PF” (proportional-fixed) null model algorithm, which reshuffles ASVs while maintaining the observed sample abundance distribution. For each group, NST values were calculated through 999 random iterations to quantify the deviation of the actual community structure from null expectations. The NST index ranges from 0% to 100%, with 50% serving as the boundary between deterministic and stochastic dominance. Values < 50% indicate that deterministic processes (e.g., environmental filtering) dominate community assembly, whereas values > 50% suggest a primary role for stochastic processes (e.g., ecological drift and random dispersal). To assess the co-occurrence relationships of fungal communities in rhizosphere soil and bulk soil, separate correlation-based co-occurrence networks were constructed for each habitat using the R package “microeco” (v1.9.0) under R version 4.4.1 (Liu et al., 2021). To reduce the interference of rare ASVs on result stability, specifically for the co-occurrence network construction, ASVs with relative abundance < 0.1% were removed from this sub-dataset prior to correlation calculation, while the remaining analyses retained the baseline dataset. Spearman rank correlation coefficients were calculated using the “WGCNA” package, and edges with |*ρ*| ≥ 0.6 and *P* < 0.01 were considered significant (Jiao et al., 2022). Network modules were identified using the cluster_fast_greedy() function in “igraph” package. Network topological properties, including Average degree, Average path length, Network diameter, Clustering coefficient, Density, Heterogeneity, Centralization, Modularity, Positive correlation (%), Negative correlation (%), were extracted using the cal_network_attr() function. Node and edge attributes were exported using the save_network() function. Networks were visualized using the Fruchterman-Reingold layout algorithm in Gephi (v0.10, https://gephi.org).

## 3 Results

### 3.1 Fungal community composition in rhizosphere and bulk soil

**Figure 2:**
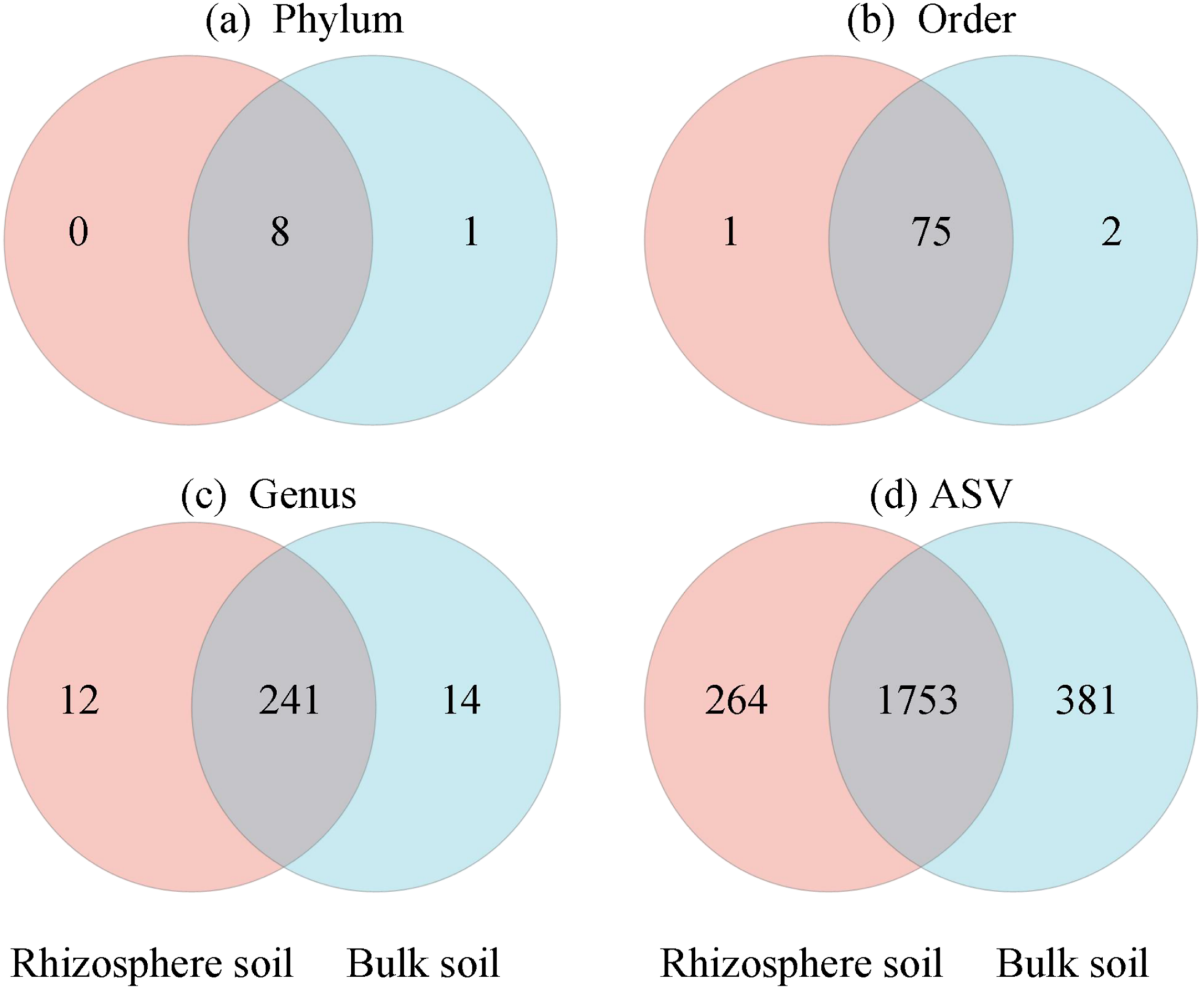
Venn diagrams showing unique and shared fungal phyla **(a)**, orders **(b)**, genera **(c)**, and ASVs **(d)** between the rhizosphere and bulk soils of *Pinus massoniana*.

A total of 5,282,069 raw sequences were obtained from the 44 samples. After quality filtering, 3,634,385 high-quality sequences remained. Downstream bioinformatics analysis and the removal of extremely rare sequences yielded a final set of 2,398 fungal ASVs. These ASVs were assigned to 9 phyla, 78 orders, and 267 genera. Venn diagram analysis revealed that the majority of fungal taxa were shared between the rhizosphere and bulk soil habitats (**Figure 2**). At the phylum level, only one phylum was unique to the bulk soil, while no phyla were exclusive to the rhizosphere (**Figure 2a**). At the order level, one and two orders were exclusively detected in the rhizosphere and bulk soil, respectively (**Figure 2b**). At the genus level, 12 genera were found only in the rhizosphere, whereas 14 were restricted to the bulk soil (**Figure 2c**). Finally, at the ASV level, 264 ASVs were unique to the rhizosphere and 381 ASVs were unique to the bulk soil (**Figure 2d**).

**Figure 3:**
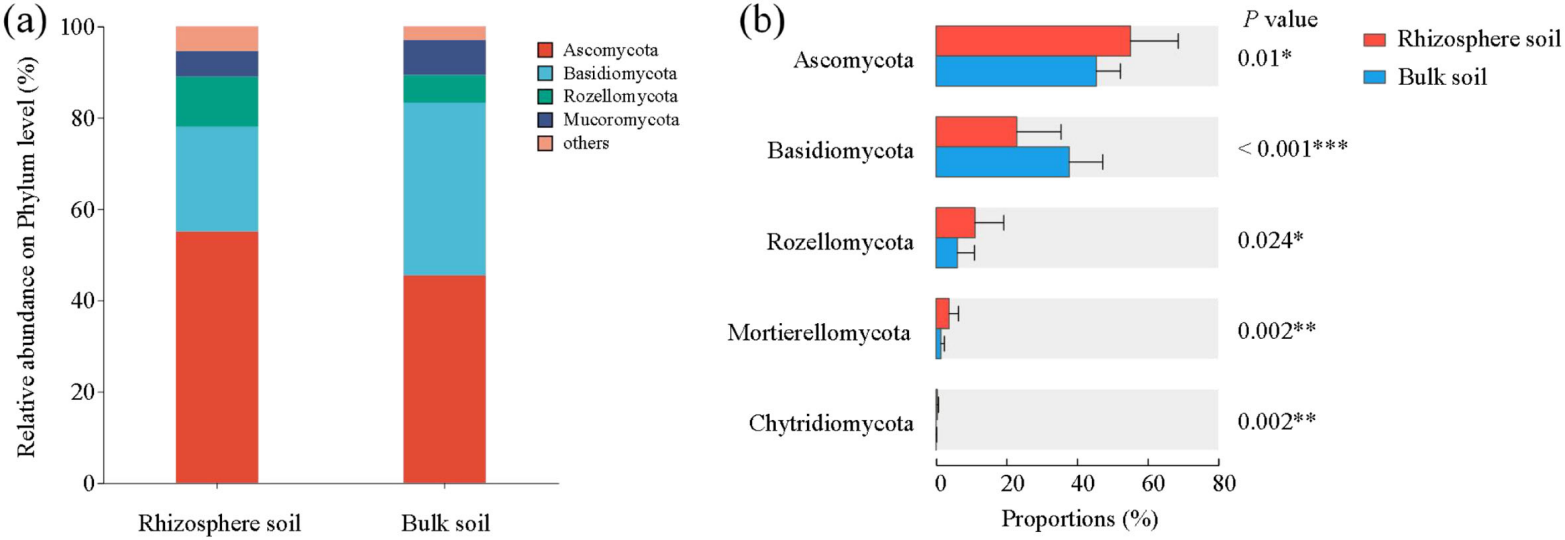
Dominant fungal phyla in the rhizosphere and bulk soils of *Pinus massoniana* **(a)**, and fungal phyla with significant differences between the two microhabitats **(b)**. Fungal phyla with relative abundance <5% are grouped as others.

*Ascomycota*, *Basidiomycota*, *Rozellomycota*, and *Mucoromycota* were the dominant fungal phyla, each accounting for more than 5% of the total relative abundance in both the rhizosphere and bulk soils (**Figure 3a**). Five fungal phyla exhibited significant differences between the two microhabitats. Specifically, the relative abundances of *Ascomycota*, *Rozellomycota*, *Mortierellomycota*, and *Chytridiomycota* were significantly higher in the rhizosphere than in the bulk soil, whereas the relative abundance of *Basidiomycota* was significantly lower in the rhizosphere than in the bulk soil (**Figure 3b**).

**Figure 4:**
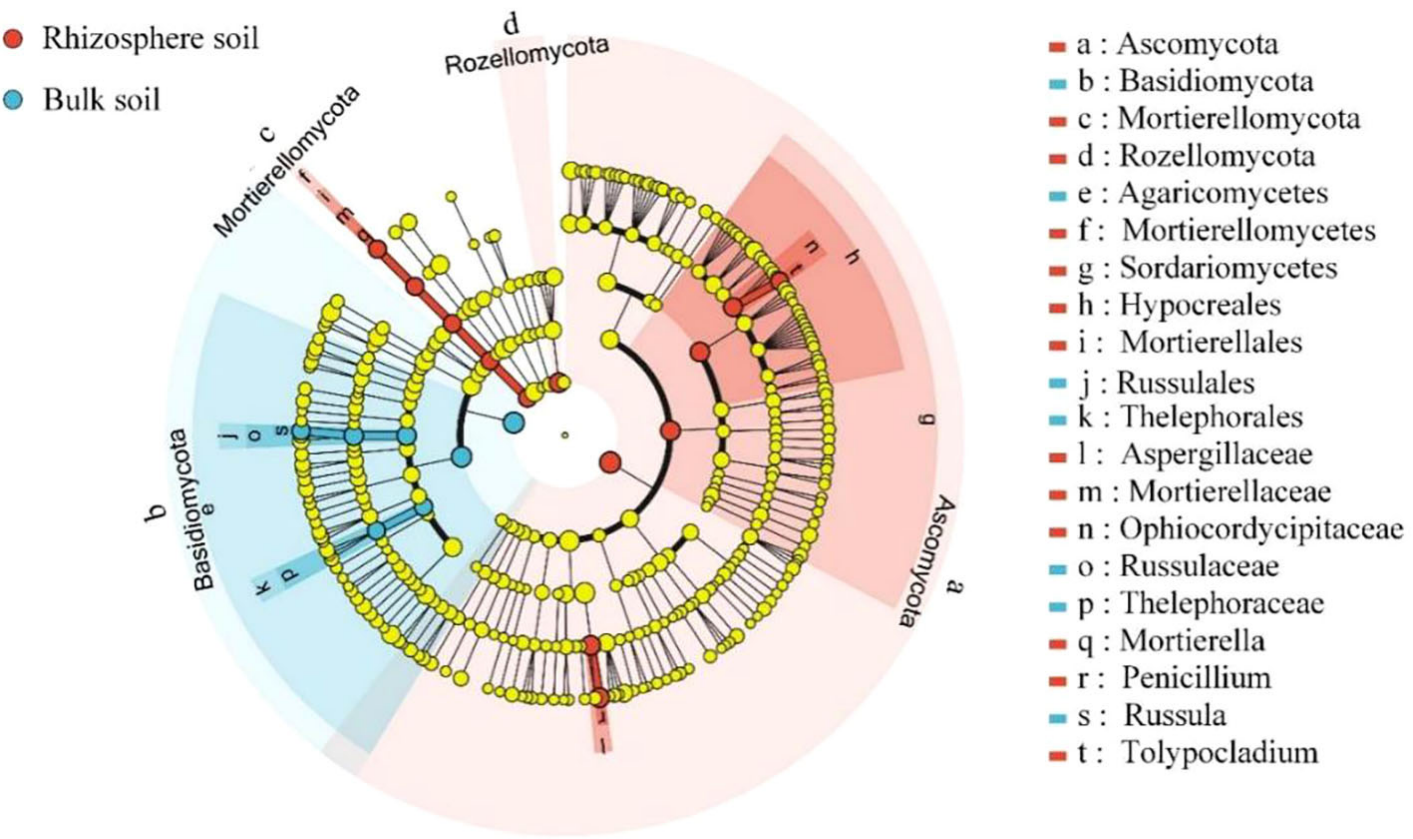
Linear Discriminant Analysis Effect Size (LEfSe) showing differences in fungal taxa between the rhizosphere and bulk soils of *Pinus massoniana*. Each concentric ring represents a taxonomic level from phylum (innermost) to genus (outermost). Nodes on the rings represent individual taxa, with node diameter proportional to their relative abundance. Taxa with significantly higher relative abundance in different microhabitats (rhizosphere or bulk soil) are highlighted in the evolutionary cladogram using colors corresponding to their respective microhabitats.

Linear Discriminant Analysis Effect Size (LEfSe) revealed 20 fungal taxa that were significantly enriched in either the rhizosphere or bulk soil, serving as potential bioindicators for these microhabitats (LDA > 4, *P* < 0.05; **Figure 4**). Specifically, the rhizosphere was significantly enriched with members of *Ascomycota*, including the class *Sordariomycetes*, the order *Hypocreales*, the families *Ophiocordycipitaceae* and *Aspergillaceae*, and the genera *Tolypocladium* and *Penicillium*. Furthermore, *Mortierellomycota* (along with its subordinate class *Mortierellomycetes*, order *Mortierellales*, family *Mortierellaceae*, and genus *Mortierella*) and *Rozellomycota* were also notably enriched in the rhizosphere. Functional guild assignment revealed that these rhizosphere-enriched genera predominantly inclined toward a benign saprophytic nature rather than a plant pathogenic profile; for instance, *Mortierella* and *Penicillium* represent highly active saprotrophic decomposers and phosphorus-solubilizers that functionally adapt to labile carbon inputs from root exudates.

In contrast, the bulk soil showed significant enrichment of Basidiomycota, primarily driven by the class *Agaricomycetes*, the orders *Russulales* and *Thelephorales*, the families *Russulaceae* and *Thelephoraceae*, and the genus *Russula* (**Figure 4**). Crucially, rather than being driven by saprotrophic or pathogenic agents, this bulk soil enrichment was characterized by symbiotic ectomycorrhizal (ECM) lineages, as represented by the genus *Russula*, which maintains extensive foraging hyphal networks within the stable mineral background pool.

### 3.2 Fungal diversity and community assembly mechanisms in rhizosphere and bulk soils

**Figure 5:**
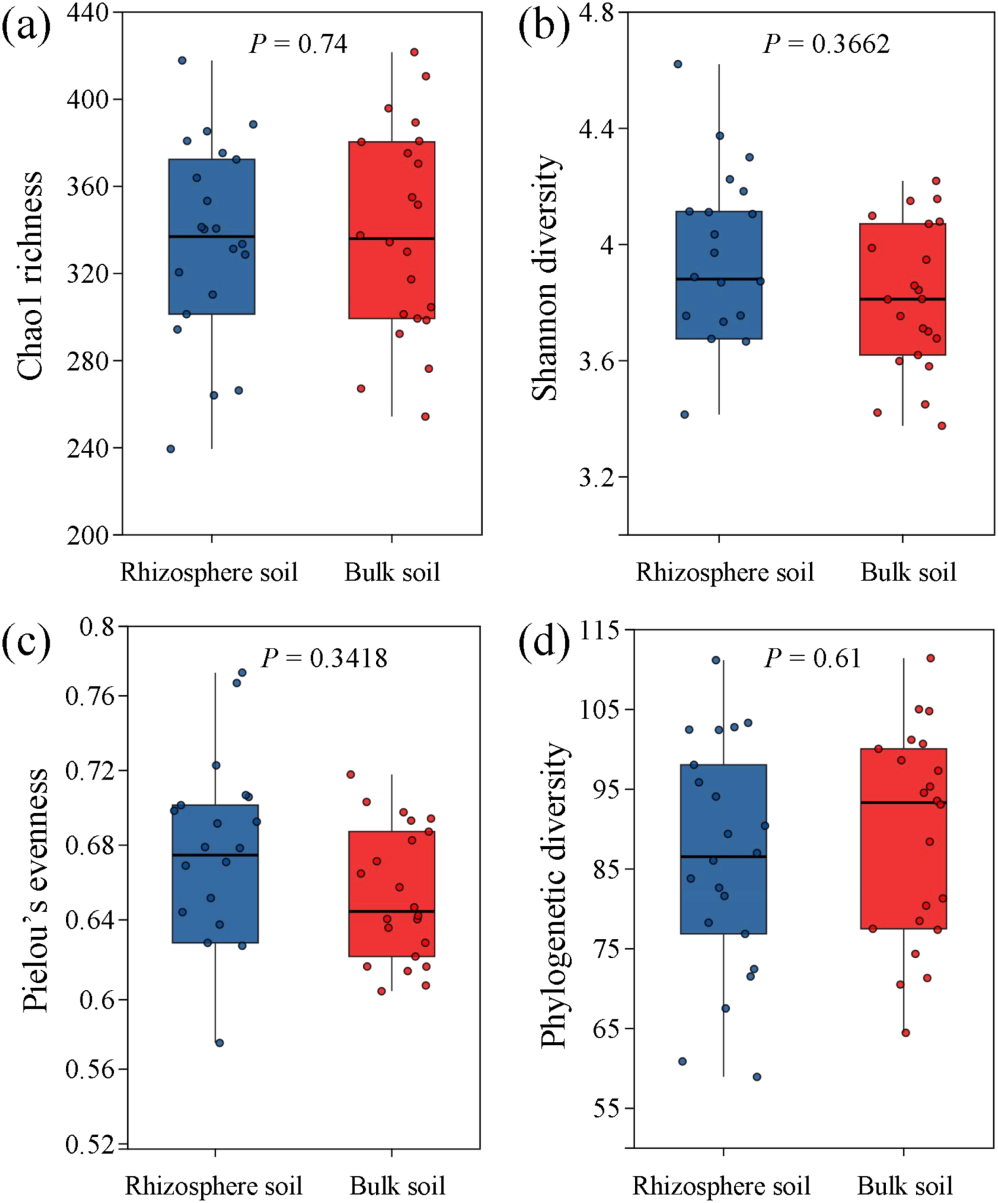
Alpha diversity of fungal communities in the rhizosphere and bulk soils of *Pinus massoniana*. **(a)** Chao1 richness; **(b)** Shannon diversity; **(c)** Pielou’s evenness; **(d)** Phylogenetic diversity.

The Chao1 richness, Shannon diversity, Pielou’s evenness, and Phylogenetic Diversity (PD) index were employed to characterize the α-diversity of fungal communities in rhizosphere and bulk soil. There were no significant differences in alpha diversity between the rhizosphere and bulk soil fungal communities for any of the tested indices (all *P* > 0.05; **Figure 5**).

**Figure 6:**
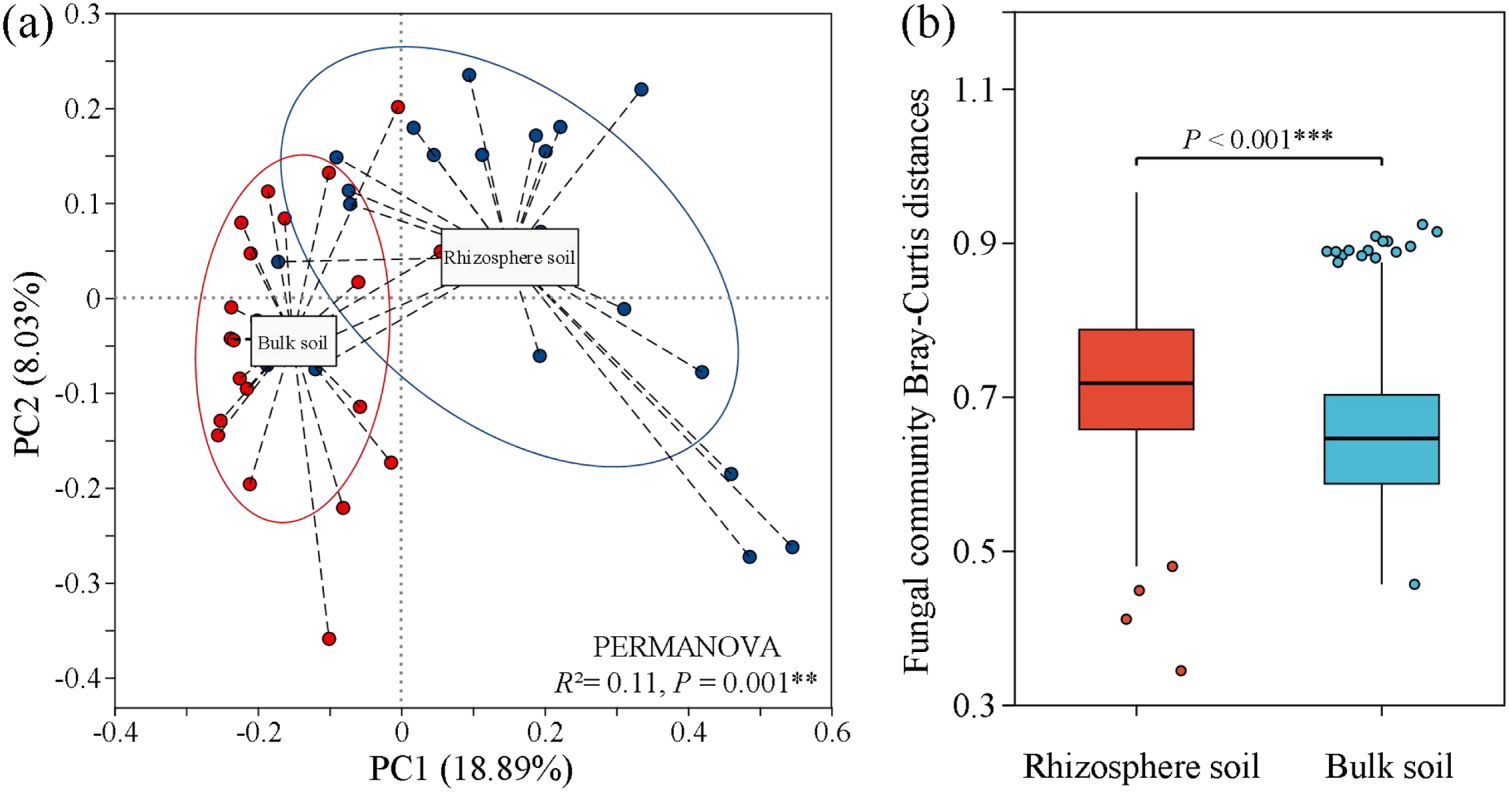
Principal coordinate analysis (PCoA) of fungal communities in the rhizosphere and bulk soils based on Bray–Curtis distance **(a)**, and dissimilarity indices of fungal communities among samples within different microhabitats **(b)**.

Principal coordinates analysis (PCoA) based on Bray–Curtis distances was performed to evaluate the structural variations in fungal communities between the rhizosphere and bulk soils. The first two principal axes of the PCoA explained 26.9% of the total variation, with the first (PC1) and second (PC2) axes accounting for 18.89% and 8.03%, respectively (**Figure 6a**). The PCoA plot revealed a distinct separation between the fungal communities of the two microhabitats. Furthermore, PERMANOVA confirmed that the fungal community structure differed significantly between the rhizosphere and bulk soils (*P* = 0.001; **Figure 6a**). Notably, the Bray–Curtis dissimilarity among samples within the rhizosphere was significantly higher than that observed within the bulk soil (*P* < 0.001; **Figure 6b**).

**Figure 7:**
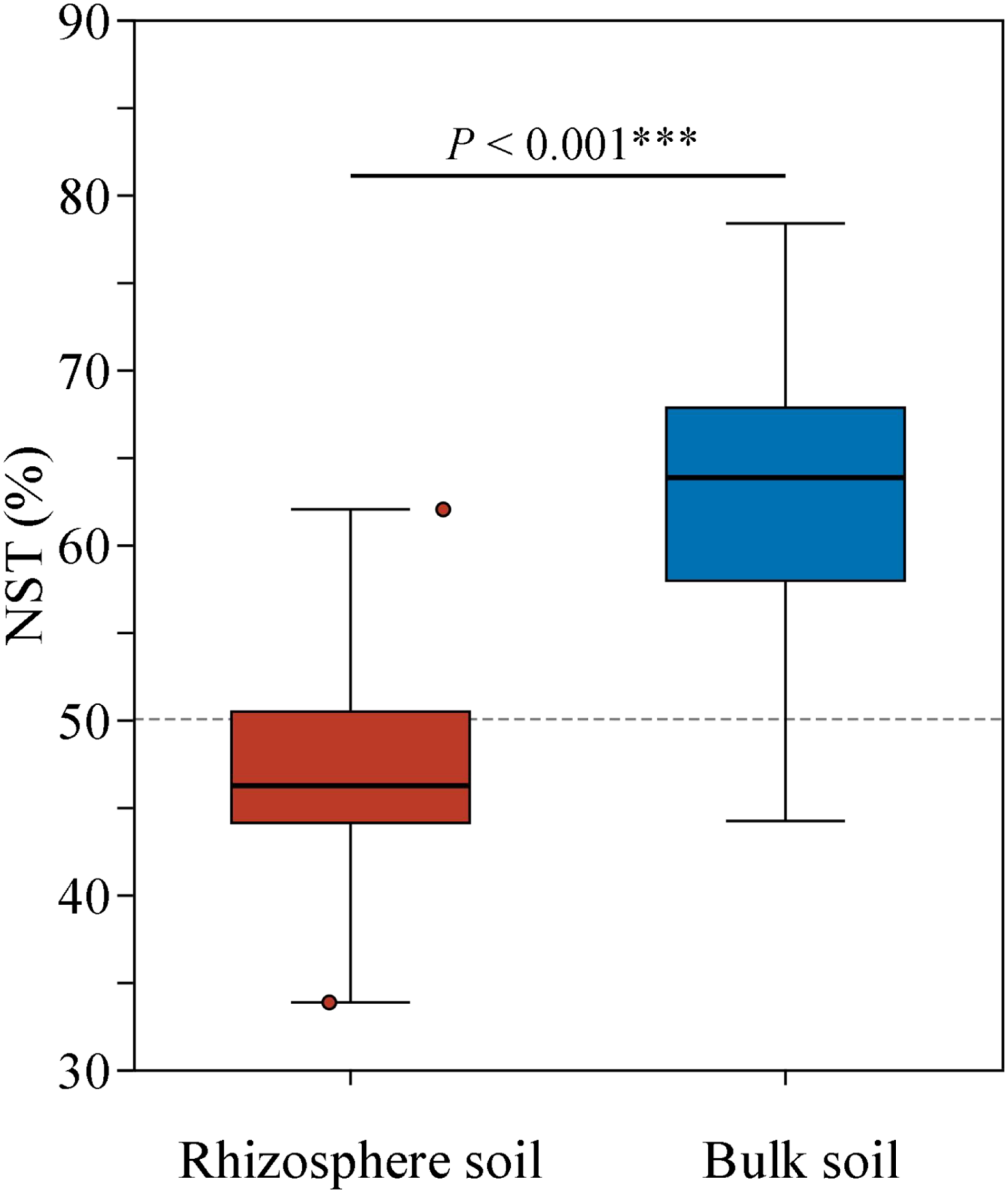
Assembly mechanisms of fungal communities in the rhizosphere and bulk soils of *Pinus massoniana* based on the normalized stochasticity ratio (NST).

The assembly mechanisms of fungal communities in the rhizosphere and bulk soils were evaluated using a null model framework. The results revealed distinct differences in the assembly processes between the two microhabitats, as evidenced by a significant difference in Normalized Stochasticity Ratio (NST) values (*P* < 0.001; **Figure 7**). In the rhizosphere soil, the mean NST value was 46.9% (i.e., < 50%), indicating that deterministic processes exerted a stronger influence than stochastic processes on fungal community assembly. In contrast, the mean NST value for bulk soil was 62.9% (i.e., > 50%), suggesting that stochastic processes played a more dominant role in shaping the fungal community structure in the bulk soil environment (**Figure 7**).

### 3.3 Fungal co-occurrence network characteristics in the rhizosphere and bulk soils

**Figure 8:**
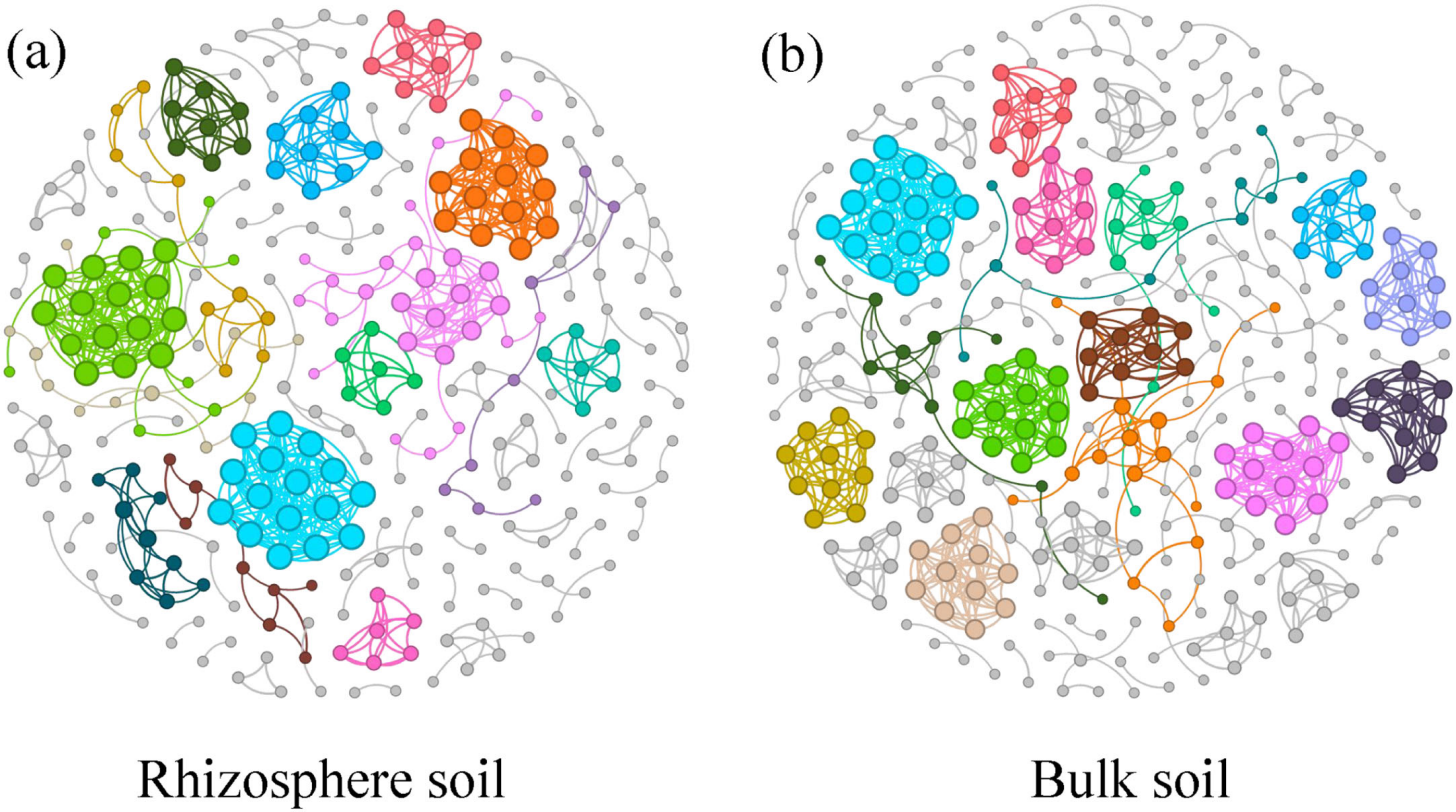
Co-occurrence networks of fungal communities in the rhizosphere **(a)** and bulk soil **(b)**. Each node represents a fungal ASV, with node size proportional to its node degree in the network. Nodes with significant correlations (Spearman’s *ρ* > 0.6, *P* < 0.01) are connected by edges. Different colors represent modules.

Fungal co-occurrence network and topological structure analyses revealed substantial structural differences between the *Pinus massoniana* rhizosphere and bulk soil fungal communities (**Figure 8**; **Table 1**). Compared to the rhizosphere, the bulk soil network exhibited a higher number of nodes (348) and edges (839), along with higher average degree and clustering coefficient, suggesting a more complex and highly connected network architecture. In contrast, the rhizosphere network showed higher levels of heterogeneity and centralization, indicating a more uneven distribution of nodal connections. Both soil types exhibited high modularity indices (> 0.9), reflecting a strong modular structure within the fungal communities. Furthermore, positive correlations were predominant in both networks, accounting for more than 99% of the total edges.

**Table 1:** Topological properties of co-occurrence networks of fungal communities in the rhizosphere and bulk soils of *Pinus massoniana*.

Topological properties of co-occurrence networks	Rhizosphere soil	Bulk soil
Nodes	312	348
Edges	683	839
Average degree	4.378	4.822
Average path length	1.958	1.23
Network diameter	7	5
Clustering coefficient	0.932	0.978
Network Density	0.014	0.014
Heterogeneity	1.002	0.843
Centralization	0.034	0.029
Modularity	0.906	0.941
Positive correlation (%)	99.71	99.52
Negative correlation (%)	0.29	0.48

## 4 Discussion

This study demonstrates that the rhizosphere and bulk soil fungal communities of *Pinus massoniana* diverge significantly in their composition, assembly mechanisms, and network architectures. The rhizosphere is a unique plant–microbe interface characterized by the continuous secretion of root exudates, such as organic acids and sugars, which create biological selection and nutrient gradients (Badri et al., 2009). In this context, the rhizosphere-enriched patterns we observed (e.g., higher relative abundance of *Ascomycota*, *Rozellomycota*, and *Mortierellomycota*) are consistent with the “rhizosphere filtration” concept proposed by Philippot et al. (2013), which suggests that plant roots actively recruit specific microbial cohorts from the surrounding soil reservoir that are capable of efficiently metabolizing rhizodeposition. This recruitment is increasingly recognized as a “metabolic coupling” process, where pre-programmed developmental changes in root exudate chemistry interact with the specific substrate preferences of soil microorganisms (Zhalnina et al., 2018). Recent evidence suggests that even volatile organic compounds and secondary metabolites such as benzoxazinoids play roles in this restructuring, acting as signaling cues to drive the assembly of host-specific microbiota (Cadot et al., 2021; Cao et al., 2025). Crucially, our prior environmental characterization of these identical *P. massoniana* plantations demonstrated that root-driven traits uniquely govern 8.8% of the rhizosphere fungal community variance, while traditional soil factors exert a minimal solo effect of only 1.3% (Lin et al., 2024). This strongly indicates that host-mediated biological selection, rather than bulk soil background chemistry, is the primary driver of this rhizosphere taxonomic enrichment. In contrast, Basidiomycota (including the orders *Russulales* and *Thelephorales*) exhibited higher relative abundance in the bulk soil. As highlighted in the global fungal survey by Tedersoo et al. (2014), many *Basidiomycota* taxa, particularly ectomycorrhizal and saprotrophic fungi, possess specialized niches in stable mineral soils, reflecting a fundamental trade-off in niche breadth between the highly dynamic rhizosphere and the relatively quiescent bulk soil. This niche specialization pattern is a common feature in forest ecosystems where host selection overrides soil background effects (Chen et al., 2019). Concurrently, this local biological selection must be interpreted within the regional context; the warm, humid subtropical monsoon climate of the study region promotes active host vegetative growth and high fine-root turnover, fueling the stable supply of labile exudates that drives this distinct rhizosphere taxonomic enrichment despite regional soil constraints.

While α-diversity indices did not differ significantly between microhabitats, *β*-diversity analysis revealed a clear separation in community structure. This pattern suggests that the rhizosphere effect in *P. massoniana* is manifested more as compositional turnover and shifts in dominance (i.e., “reorganization” of taxa) rather than changes in total richness/evenness per se. Notably, the higher Bray–Curtis dissimilarity among rhizosphere samples indicates stronger within-habitat heterogeneity near roots, which may reflect fine-scale spatial patchiness and temporal dynamics of root influence (e.g., variable exudation pulses and microsite heterogeneity), even when overall α-diversity remains unchanged. This “turnover” pattern of community variation may imply that ecosystem functioning is maintained through functional redundancy and the reorganization of core functional groups rather than total species richness (Ayala-Ortiz et al., 2025). Notably, because we did not directly quantify functional traits or metagenomic capacities, statements about functional redundancy and ecosystem functioning should be treated as hypotheses to be tested in future work. Nevertheless, the observed compositional turnover in the absence of richness changes aligns with the conceptual framework of functional insurance in microbial ecology (Lei et al., 2025). Such reconfiguration is sometimes interpreted as a host-facilitated enrichment of taxa that may contribute to nutrient mobilization or pathogen suppression (Wu et al., 2025). For instance, the rhizosphere-enriched genus *Mortierella* is well-known for its ability to solubilize phosphorus and promote plant nutrient uptake (Osorio and Habte, 2001). In our study, the enrichment of *Mortierella* supports its potential ecological relevance in the rhizosphere, but its specific functional contribution in this system requires direct functional validation.

Further NST analysis revealed that rhizosphere fungal assembly was more deterministic (NST = 46.9%), whereas bulk soil assembly was predominantly driven by stochastic processes (NST = 62.9%). This contrast is consistent with the expectation that root-influenced environments can impose stronger ecological filtering than surrounding bulk soils, thereby shifting apparent community assembly tendencies toward determinism. This supports the environmental filtering model, wherein plant-driven selection pressures may dictate a deterministic successional trajectory for the rhizosphere microbiome—a mechanism consistent across various woody plant species (Chamard et al., 2024). The transition from stochasticity in bulk soil to determinism in the rhizosphere suggests that deterministic forces can become relatively more important near roots, potentially reducing the relative role of neutral processes such as drift. This deterministic shift is highly compatible with the environmental filtering mechanisms documented in these forest stands, where the bulk soil matrix is predominantly modulated by a suite of key soil physicochemical properties—including soil bulk density, electrical conductivity, and available phosphorus—explaining 19.2% of the overall fungal variance (Chen et al., 2022). The warm and humid subtropical monsoon climate, characterized by abundant precipitation and strong seasonal rainfall, may promote host growth, fine-root turnover, and rhizosphere carbon inputs, thereby contributing to fungal enrichment and community differentiation in the rhizosphere. At the same time, regional soil physicochemical properties, including soil bulk density, electrical conductivity, available phosphorus, soil C:N ratio, nitrate nitrogen, and silt proportion, may shape the background fungal species pool and contribute to spatial heterogeneity in bulk soil fungal communities. Once entering the root zone, however, the predictable input of host rhizodeposition overrides these background edaphic variations, narrowing the ecological niches and channeling the successional trajectory toward deterministic assembly (Zhao et al., 2023). Furthermore, the assembly of such deterministic communities has been linked to enhanced sugar homeostasis and systemic resistance against soil-borne pathogens (Chen et al., 2025). Conversely, the bulk soil community appeared to be more influenced by dispersal limitation and neutral processes (Ye et al., 2023). Again, while our NST results are compatible with this interpretation, confirming dispersal limitation (as opposed to other stochastic components) would require explicit spatial or dispersal-related analyses.

This deterministic shift expands our understanding of how α- and β-diversity feed back into community assembly and downstream ecosystem functions. Although our evaluation of Chao1, Shannon, Pielou’s evenness, and phylogenetic diversity indexes revealed no significant differences in local α-diversity between the two microhabitats, our β-diversity profiling via PCoA and PERMANOVA demonstrated a highly significant, directional turnover based on Bray–Curtis distances. This combination suggests that the forest rhizosphere effect operates strictly through structural reorganization and localized niche partitioning rather than altering total species capacities. Mechanistically, this baseline α-diversity pool stabilizes population scales and buffers the community against random birth-death events. When host selective pressures are applied to this phenotypic reservoir, it accelerates deterministic environmental filtering rather than random sorting, directly explaining why the mean NST value dropped to 46.9% in the rhizosphere. Conversely, in the stochastically governed bulk soil matrix (NST = 62.9%), physical compartmentalization intensifies dispersal limitation and neutral drift, uncoupling spatial composition from immediate edaphic tracking (Zhou and Ning, 2017). From a functional perspective, the deterministic control in the rhizosphere yields a streamlined, niche-optimized consortium that secures tight “metabolic coupling” with host exudates to maximize targeted nutrient mobilization (e.g., via the enriched Mortierella cohorts). In contrast, the stochastically assembled bulk soil preserves an erratic but broader regional species repository, fundamentally functioning as a critical engine for “functional insurance” to maintain long-term ecosystem resilience against systemic environmental perturbations (Mori et al., 2018).

Fungal co-occurrence networks also displayed distinct topological features. The bulk soil network was more complex, with a higher number of nodes, edges, and average degree. Importantly, these networks are correlation-based co-occurrence patterns and do not directly demonstrate biological interactions or causal relationships; thus “positive links” should not be equated with cooperation without further validation. While high connectivity in such networks can foster community redundancy, it may also reduce the robustness of the system to environmental fluctuations, as perturbations can rapidly propagate through highly coupled nodes (de Vries et al., 2018). Given that we did not perform robustness or perturbation simulations, the stability implications of higher connectivity in bulk soil should be regarded as inferential. Both microhabitats exhibited very high modularity indices (> 0.9), indicating strongly compartmentalized structures, whereas their connectivity and connection inequality differed (i.e., bulk soil showed higher connectivity/complexity, while the rhizosphere showed higher centralization and heterogeneity). The high modularity observed in both microhabitats (> 0.9) likely reflects the inherent heterogeneity of forest soils, which limits the panmictic interaction of fungal hyphae (Deng et al., 2024; Gu et al., 2024). Furthermore, this pervasive high modularity is ecologically expected given the monospecific nature of these *P. massoniana* plantations; the high homogeneity of host-derived biotic inputs and structural properties across the site fundamentally reinforces discrete niche compartmentalization, thereby maintaining distinct sub-communities even amidst localized disturbances. Rather than being “intrinsically linked,” the co-occurrence of high modularity and more deterministic assembly in the rhizosphere is best viewed as compatible with strong environmental filtering that can promote non-random compartmentalization, pending formal tests of process–topology relationships. High modularity is often considered a hallmark of stable microbial systems, as it partitions functional roles and confines localized disturbances, thereby preventing systemic network collapse (Gu et al., 2024). According to the network theory established by Olesen et al. (2007), high modularity limits the spread of localized disturbances, thereby enhancing the overall stability of the microbial community. In our dataset, we did not explicitly identify network keystone taxa (e.g., via within-/among-module connectivity roles), so statements about specific taxa being “keystone” should be framed as hypotheses pending targeted network role analysis and experimental verification. Accordingly, while *Mortierella* was enriched in the rhizosphere and is ecologically meaningful in prior studies, its hub/keystone status in our rhizosphere network remains to be tested rather than concluded (Bei et al., 2025). We therefore emphasize network-level contrasts in organization (complexity vs centralization/heterogeneity) rather than assigning functional “keystone” roles to particular taxa without role analysis. This architectural difference highlights that while domestication or cultivation can simplify microbial networks, wild-type or forest-based systems like *P. massoniana* maintain modular integrity to sustain ecological functions (Deng et al., 2024).

Overall, our results provide evidence that rhizosphere *vs* bulk soil habitats are associated with distinct fungal community composition, different NST-based assembly tendencies, and contrasting co-occurrence network topology in *P. massoniana*. By pairing these high-resolution biological organization patterns with our previously established edaphic baselines, this study underscores a clear partition of ecological drivers at the forest plant–soil interface. Future work integrating soil physicochemical profiling, root trait/exudate measurements, and functional assays (or meta-/metatranscriptomics), together with network robustness analyses across prolonged temporal scales (e.g., multiple seasons or forest age gradients), will be essential to identify key drivers and evaluate the functional consequences implied by these patterns.

## 5 Conclusion

This study systematically characterized the multi-dimensional heterogeneity between the rhizosphere and bulk soil fungal communities of *P. massoniana*. Our findings lead to the following key conclusions: First, the rhizosphere fungal community exhibited distinct structural features across multiple taxonomic levels (phylum, order, and genus), with a significant enrichment of taxa such as *Ascomycota* and *Mortierellomycota*, suggesting stronger microhabitat filtering in the root-influenced zone. Second, despite the lack of significant differences in α-diversity, fungal composition (*β*-diversity) and ASV abundance patterns showed clear divergence, indicating that the rhizosphere effect is expressed primarily as compositional turnover and shifts in dominance rather than changes in overall richness/evenness. Third, the community assembly mechanisms differed fundamentally, with deterministic processes dominating the rhizosphere and stochastic processes prevailing in the bulk soil, supporting a shift toward relatively stronger deterministic assembly near roots as inferred by NST. Finally, co-occurrence network analysis revealed that the rhizosphere community formed a compact but highly centralized network, suggesting a more centralized and heterogeneous network organization, whereas the bulk soil network was more complex and decentralized. Notably, co-occurrence networks are correlation-based and do not directly imply interactions or functional regulation; thus, functional interpretations should be treated cautiously. In summary, *P. massoniana* rhizosphere and bulk soil habitats are associated with distinct fungal community composition, different NST-inferred assembly tendencies, and contrasting network organization strategies. These results provide comparative evidence for understanding rhizosphere-driven community differentiation in subtropical conifer forest soils and offer reference information for future work integrating environmental measurements and functional assays to evaluate ecological implications and management relevance.

## Data Availability

The datasets presented in this study can be found in online repositories. The names of the repository/repositories and accession number(s) can be found in the article/supplementary material.
